# Biomarkers in breast cancer 2024: an updated consensus statement by the Spanish Society of Medical Oncology and the Spanish Society of Pathology

**DOI:** 10.1007/s12094-024-03541-1

**Published:** 2024-06-13

**Authors:** Ramon Colomer, Blanca González-Farré, Ana Isabel Ballesteros, Vicente Peg, Begoña Bermejo, Belén Pérez-Mies, Susana de la Cruz, Federico Rojo, Sonia Pernas, José Palacios

**Affiliations:** 1 UAM Personalised Precision Medicine Chair & Medical Oncology Department, La Princesa University Hospital and Research Institute, C/Diego de León, 62, 28006 Madrid, Spain; 2 Pathological Anatomy Service, Clínic Hospital, Barcelona, Spain; 3 Medical Oncology Department, La Princesa University Hospital, Madrid, Spain; 4 Pathological Anatomy Service, Vall d’Hebron University Hospital, Barcelona, Spain; 5 Medical Oncology Department, Biomedical Research Institute INCLIVA, Medicine Department of the University of Valencia and Clinic University Hospital, Valencia, Spain; 6 Pathological Anatomy Service, Ramón y Cajal University Hospital, Faculty of Medicine, University of Alcalá, IRYCIS and CIBERONC, Madrid, Spain; 7 Medical Oncology Department, Navarra University Hospital, Navarre, Spain; 8 Anatomy Service, Fundación Jiménez Díaz University Hospital and CIBERONC, Madrid, Spain; 9 Oncology Department, Catalan Institute of Oncology (ICO)-IDIBELL, L’Hospitalet, Barcelona, Spain; 10 Pathological Anatomy Service, Department of Pathology, Ramón y Cajal University Hospital, Faculty of Medicine, University of Alcalá, IRYCIS and CIBERONC, Ctra. Colmenar Viejo, Km 9,1, 28034 Madrid, Spain

**Keywords:** Breast neoplasm, Diagnostic, Gene expression profiling, Prognostic, Therapy predictive

## Abstract

This revised consensus statement of the Spanish Society of Medical Oncology (SEOM) and the Spanish Society of Pathological Anatomy (SEAP) updates the recommendations for biomarkers use in the diagnosis and treatment of breast cancer that we first published in 2018. The expert group recommends determining in early breast cancer the estrogen receptor (ER), progesterone receptor (PR), Ki-67, and Human Epidermal growth factor Receptor 2 (HER2), as well as BReast CAncer (*BRCA*) genes in high-risk HER2-negative breast cancer, to assist prognosis and help in indicating the therapeutic options, including hormone therapy, chemotherapy, anti-HER2 therapy, and other targeted therapies. One of the four available genetic prognostic platforms (Oncotype DX^®^, MammaPrint^®^, Prosigna^®^, or EndoPredict^®^) may be used in ER-positive patients with early breast cancer to establish a prognostic category and help decide with the patient whether adjuvant treatment may be limited to hormonal therapy. In second-line advanced breast cancer, in addition, phosphatidylinositol-4,5-bisphosphate 3-kinase catalytic subunit alpha (PIK3CA) and estrogen receptor 1 (ESR1) should be tested in hormone-sensitive cases, *BRCA* gene mutations in HER2-negative cancers, and in triple-negative breast cancer (TNBC), programmed cell death-1 ligand (PD-L1). Newer biomarkers and technologies, including tumor-infiltrating lymphocytes (TILs), homologous recombination deficiency (HRD) testing, serine/threonine kinase (AKT) pathway activation, and next-generation sequencing (NGS), are at this point investigational.

## Introduction

Biomarker analysis in cancer provides information that complements classical clinical factors, and also enables certain treatments in patients to be selected [1]. In breast cancer, biomarker analysis began with testing for hormone receptor expression to guide tamoxifen therapy. The subsequent inclusion of targeted treatments against human epidermal growth factor receptor 2 (HER2) revolutionized the biomarker field. It also highlighted that biomarker test methods need to be standardized and harmonized. The intervening years have also seen progress in the understanding of single molecular abnormalities in breast cancer related to specific molecular therapies, such as BReast CAncer (*BRCA*) gene, programmed cell death-1 ligand (PD-L1), phosphatidylinositol 3-kinase (PI3K), or estrogen receptor 1 (ESR1). The clinical potential for monitoring disease using new technologies grouped under the term liquid biopsy is currently being studied.

The purpose of these revised consensus guidelines from the Spanish Society of Medical Oncology (SEOM) and the Spanish Society of Pathological Anatomy (SEAP) is to recommend which biomarkers should routinely be tested in patients with breast cancer; including conventional markers, genetic platforms, and newer technologies, as well as those that remain investigational. Recommendations are presented in a stratified fashion, depending on whether the breast cancer is in an early or advanced stage.

## Early-stage breast cancer

### Histological type and grade

Histological typing should be performed according to World Health Organization (WHO) criteria. Among luminal carcinomas, pure tubular, cribriform and mucinous carcinomas have better prognosis than invasive carcinomas of no special type (IC-NST). It is controversial whether invasive lobular carcinoma has a different prognosis than IC-NST, but an accurate diagnosis of this tumor type, based on both morphological features and E-cadherin expression pattern, is recommended, especially considering the specific clinicopathological and molecular features of this histological type. Among triple-negative tumors (TNBC), histological types of good prognosis include adenoid cystic carcinoma, secretory carcinoma, and other salivary gland-type tumors. In addition, fibromatosis-like and low-grade adenosquamous carcinomas are two types of metaplastic carcinomas with good prognosis. In contrast, high-grade metaplastic carcinomas (spindle cell, squamous, and matrix-producing carcinomas) have a worse prognosis and less response to chemotherapy than other TNBC.

Histological grade has independent prognostic value at all stages of breast cancer. Therefore, all invasive breast carcinomas, irrespective of their histological type, should be graded following a protocolized method [1]. The WHO classification recommends using the Nottingham (Elston-Ellis) modification of the Patey–Scarff and Bloom–Richardson grading system. Grading is evaluated by a numerical scoring system of 1–3 per category (tubular formation, nuclear pleomorphism, and mitotic count). Only clear central lumina enclosed by polarized cells should be counted for tubular/gland formation. Nuclear pleomorphism is scored in the least differentiated tumoral area. Mitotic count is performed in the most proliferative area, typically at the periphery of the tumor. This parameter has been reported as the most important constituent of grade, so only clear mitosis should be counted. The final score should be adapted to the high-power field size of the microscope used [2]. When these recommendations are strictly followed the inter-observer agreement level is high, and they can be applied to tissue obtained by core-needle biopsy [1]. In the future, artificial intelligence algorithms may be a helpful tool for improving reproducibility or even automatically grading breast carcinomas [3].

### Hormone receptors

Estrogen receptor (ER)-alpha and progesterone receptor (PR) status must be determined in all newly diagnosed breast carcinomas, as well as in metastatic or recurrent tumors [4].

ERs are expressed in about 70% of invasive breast carcinoma cases. ER is a strongly predictive factor of a response to hormone therapy as well as a favorable prognostic factor [5]. In the 2020 ER and PR guidelines from the American Society of Clinical Oncology and the College of American Pathologists (ASCO-CAP), the cut-off that indicates patients who will benefit from endocrine therapy remains at 1% of cancer nuclei stained for ER, irrespective of staining intensity. About 2–3% of breast carcinomas will have 1–10% cell staining for ER. This group represents a clinical challenge, not only for the low reproducibility of the results between laboratories, but also for the real benefit of antiestrogenic therapy for these patients (these tumors seem more related at the molecular level to ER-negative tumors). In this sense, the ASCO-CAP guidelines divide the ER-positive result into positive >10% and low positive 1–10%, deciding the best treatment on the complete information about an individual case [4]. Currently, immunohistochemistry (IHC) is recommended as primary screening. Only nuclear positivity in tumoral cells is scored, avoiding normal breast tissue from being mixed with the tumor. Both staining intensity and the percentage of positive cells are recorded. Alternatively, a score can be reported, like the one described by Allred, et al., combining the estimated nuclear positivity rate in cancer cells (a score of 0–5, based on the percentage) with staining intensity (intensity 0–3) [6]. It is useful to test for ER-alpha in ductal carcinoma in situ because hormone suppression treatment can reduce the recurrence risk by 50% in ER-positive patients [7].

PR is expressed in about 60% of cases of invasive ductal carcinoma of the breast. In general, correlation between ER-alpha and PR expression is good, although 10% of cases may prove to be ER-alpha-positive and PR-negative. These patients have a higher risk of recurrence than ER-alpha-positive, PR-positive cases [8, 9]. Fewer than 5% of patients may prove to be PR-positive, ER-negative. The methodology and quantification used are the same as for ER-alpha, with cut-off in 1% of stained nuclei. Recent studies suggest that PR expression <20% might have adverse prognostic implications [9]. PR determination in in situ carcinoma is optional [4].

Two-thirds of breast cancers express androgen receptors (ARs). Their role in carcinogenesis and as a novel therapeutic target has been explored using antiandrogens either alone or in combination, with some promising yet limited clinical results [10, 11]. Current guidelines do not include AR determination as a routine biomarker for clinical practice [12].

### HER2 assessment

HER2 must be analyzed for overexpression or amplification in all breast carcinomas, whether early or advanced. HER2 amplification is a predictive factor for anti-HER2 therapies and an unfavorable prognostic factor, when not treated. The use of anti-HER2 antibodies in combination with chemotherapy or hormonal therapy has dramatically improved the clinical course of HER2-positive breast cancer. A better understanding of tumor biology and HER2 signaling has led to the development of new strategies to further improve patient outcomes. Current novel HER2-targeted therapies include dual-HER2 inhibition with monoclonal antibodies, such as trastuzumab plus pertuzumab; antibody–drug conjugates such as trastuzumab emtansine or trastuzumab-deruxtecan (T-DXd); and tyrosine kinase inhibitors such as lapatinib, tucatinib or neratinib. The measurement and definition of HER2 amplification or overexpression have also been optimized over the years [13].

HER2 status is routinely assessed using a combination of IHC to evaluate HER2 protein expression levels and in situ hybridization (ISH) to assess *HER2* gene status. Several HER2 test methods are valid, provided the technology is standardized according to the manufacturer’s instructions, and supported by an external quality-control program [14]. There is a high concordance (98–99%) between HER2 results in core biopsies and surgical specimens, and a core biopsy sample is often the material of choice for HER2 determination [15], since the availability of pre-treatment biomarkers status allows clinicians to treat patients with the most appropriate neoadjuvant therapy (NAT) and provide important prognostic and biological information. Optimal tissue handling requirements are of primary importance and fixation time should not exceed 72 h. Importantly, in cases when pre-analytical conditions could not be guaranteed, this should be specified in the pathology report. The current ASCO-CAP recommendations propose that the HER2 test be repeated on the excision specimen if there are concerns about discordance between histopathologic findings [14, 16]. The indications of possible HER2 test discordance are specified in detail in the guidelines [16]. Of note, it is not mandatory to retest grade 3 tumors in the absence of other clinical-pathological criteria.

**Fig. 1:**
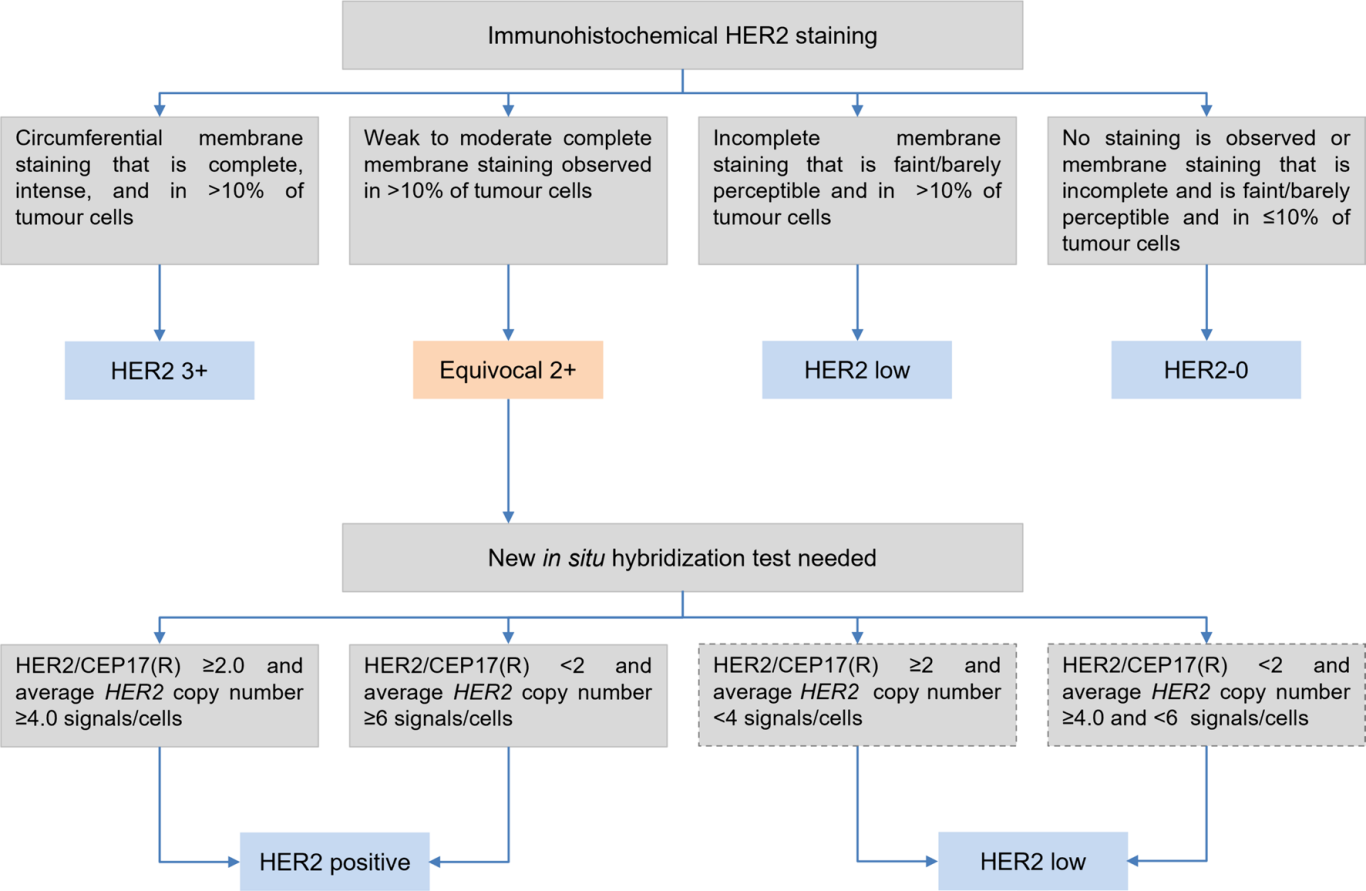
Algorithm for HER2 assessment in infiltrating breast carcinoma with an initial IHC approach. CEP17: chromosome enumeration probe 17; HER2: human epidermal growth factor receptor 2; IHC: immunohistochemistry. Results framed in dashed lines should be accompanied with a comment

*HER2* expression should be interpreted according to the 2023 ASCO-CAP guidelines [17], slightly modified in Fig. 1, which includes staining intensity, percentage of cells and staining localization [16]. However, some specific scenarios are taken into consideration. For instance, when moderate to intense basolateral or lateral membrane IHC staining is detected (a pattern frequently observed in micropapillary carcinomas), or circumferential membrane IHC staining that is intense but within ≤10% of tumor cells (heterogeneous but very limited) is observed, it is preferable to consider those cases as equivocal (2+) and a new test is desirable.

In relation to ISH tests, those laboratories using single-probe ISH assays should be encouraged to include concomitant IHC review as part of the interpretation. This concomitant review should be performed in the same institution to ensure parallel interpretation and quality of the two assays. The current diagnostic approach also includes more rigorous interpretation criteria for dual-probe ISH assay and requires concomitant IHC review in certain scenarios to come to the most accurate HER2 status designation. Those scenarios include an average *HER2* copy number <4.0 signals/cell with a *HER2*/CEP17 ratio ≥2.0 or an average *HER2* copy number ≥6.0 signals/cell or ≥4.0 and <6.0 signals/cell with a *HER2*/CEP17 ratio <2.0. If the concomitant interpretation of the IHC and ISH techniques in these three scenarios is a final negative result, a comment should be added in the final report to clarify the absence of robust evidence supporting the use of anti-HER2 therapies (Fig. 1). Importantly, if a patient develops a recurrence or metastatic disease, a new HER2 test should be performed if a tissue sample is available.

*HER2* intratumoral heterogeneity has been described in up to 40% of breast carcinomas, especially in *HER2* equivocal or *HER2* borderline cases, or chromosome 17 polysomy. The presence of *HER2* heterogeneity has been associated with a worse prognosis and lesser anti-HER2 treatment response [18].

According to current guidelines, classifying breast tumors as HER2 IHC-3+ or with *HER2* gene amplification assessed by ISH is the primary predictor of responsiveness to HER2-targeted therapies. For some recently developed anti-HER2 therapies, *HER2* expression may be a continuous variable in terms of treatment effect. Tumors with IHC-1+ or 2+ and with a negative ISH result (until now reported as HER2-negative) might be re-defined as HER2-low, with HER2 negativity being limited to IHC-0 (Fig. 1). Based on this definition, up to 55% of breast cancers are HER2-low, comprising a majority of hormone receptor-positive (HR-positive) tumors (65–83%) with different intrinsic subtypes [13, 19]. Although it has been shown that HER2-low tumors do not benefit from adjuvant trastuzumab [20], some HER2-directed antibody–drug conjugates such as T-DXd are effective in HER2-positive disease and also in HER2-low tumors with no amplification. In a randomized clinical trial performed in 557 HER2-low previously treated metastatic breast cancers, T-DXd resulted in significantly longer progression-free survival (PFS) and overall survival (OS) than the physician’s choice of chemotherapy [21]. Although primary metastatic breast cancer had a significantly lower HER2-low discordance rate than secondary metastatic breast cancer, it has been observed that relevant HER2 discordance rates are observed between different metastatic sites and molecular subtypes, therefore highlighting the importance of evaluating potentially therapy-relevant HER2-low discordance rates between a primary tumor and corresponding distant metastases [22]. Regarding the new HER2-low scenario, the 2023 ASCO-CAP guidelines do not recommend changing reporting terminology for lower levels of HER2 IHC expression (e.g., HER2-low), while the 2023 ESMO expert consensus statements of HER2-low recommend classifying all levels of HER2 expression [23]. However, the score 0 versus 1+ must be informed in all HER2-negative cases, to ensure the eligibility criteria for T-DXd therapy. The guidelines also provide best practices for discrimination of IHC 0 versus 1+.

Neratinib is a kinase inhibitor indicated for the extended adjuvant treatment of adult patients with early-stage ER-positive HER2-overexpressed/amplified breast cancer, to follow adjuvant trastuzumab-based therapy, when there is a high risk of relapse (node-positive disease) [24].

### Ki-67

Ki-67 is a nuclear protein found in all phases of the cell cycle except G0. Ki-67 expression is related, although not completely, to the histological grading of breast carcinomas. Immunohistochemical assessment of Ki-67 is the method most widely used to determine the proliferative activity of breast cancer, although the reproducibility of the results between laboratories has been disputed [12].

Calibrating the method in different laboratories substantially increases the concordance between results [25]. An international Ki-67 working group in breast cancer developed a website (https://www.ki67inbreastcancerwg.org/) that includes an app for scoring Ki-67 more accurately [26]. Briefly, to score Ki-67, any cells with any degree/intensity of brown nuclear staining are considered positive. The whole slide should be evaluated, estimating the percent area with negligible, low, medium, or high Ki-67 index. A hundred negative or positive nuclei in each field type should be counted and finally a “weighted global score” recorded for that slide [25].

Cut-off point selection for clinical application remains a controversial matter. Given the lack of standardization, both the 2021 St. Gallen consensus [27] and the Ki-67 working group [28] consider that only very low (<5%) or very high (>30%) values can be reliably categorized as low or high proliferation by visual scoring of Ki-67 IHC in routine clinical practice. In this way, in ER-positive early breast cancer with Ki-67 between 6 and 29%, a multi-parameter gene expression assay has been recommended to help in guiding adjuvant treatment [27].

Other potential uses for Ki-67 include prediction of responsiveness/resistance to chemotherapy or endocrine therapy, estimation of residual risk in patients on standard therapy and dynamic biomarker of treatment efficacy in samples taken before, during, and after NAT, particularly neoadjuvant endocrine therapy identifying patients who can be spared intensive chemotherapy in the adjuvant setting [29–31].

In advanced breast cancer, Ki-67 has also shown relevance in predicting response to cell cycle inhibitors. PFS in patients undergoing endocrine therapy plus CDK4/6i was inversely correlated with Ki-67 expression, suggesting that tumor proliferation has a great impact on cell cycle inhibitors combined with endocrine therapy [32].

Ki-67 is a useful prognostic tool that, in combination with other clinical factors, has a value comparable to that of more complex gene expression analyses [33]. The systematic use of digital imaging analysis will improve its reproducibility and value in the coming years [34].

### Genetic platforms for prognosis and chemotherapy guidance

Gene expression signatures can be helpful in deciding whether to use adjuvant chemotherapy in early breast cancer. Several retrospective studies have suggested the clinical utility of genomic signatures, although only Oncotype DX^®^ and MammaPrint^®^ are supported by prospective randomized trials (Table 1). These signatures provide different information depending on the clinical setting and are not interchangeable. The Oncotype DX^®^ trial was validated with level 1A evidence for prognosis and predicts the benefit of adjuvant chemotherapy in node-negative, ER-positive, HER2-negative early breast cancer (post- or pre-menopausal) and in node-positive post-menopausal cases. MammaPrint^®^ has level 1A prognostic evidence in node-negative, ER-positive, HER2-negative clinically high-risk breast carcinoma and level 1A evidence for determining prognosis in node-positive disease. Further clinical evidence will clarify the use of multigene testing in the node-positive setting. In addition, clinicians should be aware of the clinical utility and limitations when applying such tests, particularly since some authors have suggested that molecular testing to deliver personalized chemotherapy risks over- and under-treatment [35].

**Table 1:** Recommendations for the prognostic and predictive value of different genetic tests in breast cancer

Recommendations	Oncotype DX^®^	EndoPredict^®^	Prosigna^®^	MammaPrint^®^
NICE 2018 [36]	Prognosis: ER+, HER2−, N0/N1 (pre/post-menopausal) Prediction: ER+, HER2−, N0	Prognosis: ER+, HER2−, N0/N1 (pre/post-menopausal)	Prognosis: ER+, HER2−, N0/N1 (post-menopausal)	Not cost-effective
St Gallen 2019 [37]	Highly recommended for T1–T3 N0 stages Generic high recommendation for the use of CT in TxN1 stages	Generic recommendation for the use of CT in T1–T3 N0 and TxN1 stages	Generic recommendation for the use of CT in T1–T3 N0 and TxN1 stages	Generic recommendation for the use of CT in T1–T3 N0 and TxN1 stages
ESMO 2019 [38]	Evidence: 1A ER+, HER2−, N0/N1	Evidence: 1B ER+, HER2−, N0/N1	Evidence: 1B ER+, HER2−, N0/N1	Evidence: 1A ER+, HER2−, N0/N1
AJCC 2019 [39]	Evidence: 1	Evidence: 2	Evidence: 2	Evidence: 2
ASCO 2019 [40]	Evidence: High ER/PR+, HER2−, N0	Evidence: Intermediate ER/PR+, HER2−, N0	Evidence: High ER/PR+, HER2−, N0	Evidence: High For high-risk patients
NCCN 2021 [41]	Prediction:Yes Prognosis: Yes Evidence 1 (post-menopausal) Evidence 2A (pre-menopausal)	Prediction: No Prognosis: Yes Evidence: 2A	Prediction: No Prognosis: Yes Evidence: 2A	Prediction: No Prognosis: Yes Evidence: 1

*CT* chemotherapy, *ER* estrogen receptor, *HER2* human epidermal growth factor receptor 2, *PR* progesterone receptor

#### Oncotype DX^®^

Oncotype DX^®^ tests the expression of 21 genes and calculates a Recurrence Score (RS). Oncotype DX^®^ methodology has been optimized for application to formalin-fixed tissue, and its results have had a proven impact on treatment decisions [42]. The value of Oncotype DX^®^ for predicting the benefit provided by chemotherapy and hormone therapy in these risk groups has been examined in various studies, involving both node-negative and node-positive patients [43, 44]. The RS defines three prognostic groups. The 10-year distant recurrence rate in the low RS group is 7%, 14% in the intermediate RS group, and 30% in high RS patients. Results of two prospective trials support recommendations for treatment considering RS and menopausal age/state. TAILORx (Trial Assigning Individualized Options for Treatment [Rx]) was a prospective trial designed to determine the prognosis of a group of patients who had undergone surgery for ER-positive, HER2-negative, node-negative breast cancer with an RS of 11–25 [45]. At a median follow-up of 9 years, the hazard ratio (HR) for the endocrine group versus the chemoendocrine group was 1.08 (95% confidence interval [CI], 0.94–1.24), and the distant recurrence rate was 5%, regardless of chemotherapy administration, establishing that at age < 50 and RS 16–25, benefit can be obtained from the use of chemotherapy, whereas at age > 50 and RS < 25, there is no benefit from chemotherapy. Results from the RS < 11 group reported a 0.7% risk of distant recurrence and a 1.3% risk of any other recurrence. These results were confirmed in the Surveillance Epidemiology and End Results (SEER) database registry [46]. It shows the benefit of chemotherapy in pre-menopausal women with low RS (≤25) and 1–3 axillary lymph node involvement, with a median follow-up of 5 years (HR 0.81; 95% CI, 0.67–0.98). Results showed a 46% decrease in invasive disease-free survival and a 53% decrease in deaths, leading to an absolute improvement in OS at 5 years of 1.3%. Post-menopausal women with RS 0–25 did not benefit from adjuvant chemotherapy in any subgroup [47].

#### MammaPrint^®^

MammaPrint^®^ is a 70-gene signature test of prognostic value that classifies breast cancer patients into high-risk and low-risk groups [48]. Initially, the test required fresh tissue, although now it is optimized on formalin-fixed paraffin-embedded (FFPE) samples. MammaPrint^®^ was validated for early luminal breast cancer in node-negative patients in the RASTER trial. Its clinical utility has been demonstrated in the prospective randomized phase III MINDACT trial, performed in patients with either negative lymph nodes or 1–3 positive lymph nodes. Groups with discrepant genomic and clinical risks were randomized to receive endocrine therapy versus adjuvant chemotherapy and endocrine therapy, revealing that high-risk and low-risk patients had limited therapeutic benefit from the use of adjuvant therapy. Overall, MammaPrint^®^ has not achieved predictive utility, but it is the only multigenomic trial with prospective 1A evidence level for evaluating prognosis in high-risk patients with node-negative and node-positive early luminal breast cancer. The WSG-PRIMe study has prospectively demonstrated the impact of MammaPrint^®^ and BluePrint on treatment decision [49]. BluePrint is an 80-gene assay that allows a molecular sub-classification into low-risk luminal tumors, high-risk luminal tumors, HER2 and basal type.

#### Prosigna^®^ (PAM50/ROR)

Prosigna^®^ (NanoString Technologies, Inc.) is a second-generation multigene signature, which includes 50 genes based on NanoString nCounter technology, approved to estimate the risk of distant relapse in early ER-positive breast cancer with up to 3 positive lymph nodes in post-menopausal women treated with endocrine therapy alone [50]. PAM50 can be performed on FFPE samples locally and provides a proliferation-based measure of gene expression that, combined with node status and tumor size, defines a risk score called risk of recurrence (ROR). ROR is divided into three risk groups: low (<10%), intermediate (10–20%) and high (>20%) ROR. It correlates with the probability of distant recurrence at 10 years. Prosigna^®^ also identifies molecular subtypes. It has been validated as a prognostic tool in the ABCG-8 and TransATAC trials, in patients treated with endocrine therapy, with ER-positive and node-negative disease, although prospective data on its predictive value are needed, which will be generated in the OPTIMA study in node-positive early breast cancer [51].

#### EndoPredict^®^ (EPclin)

EndoPredict^®^ (Myriad Genetics, Inc.) is a 12-gene prognostic test that estimates 10-year relapse risk and provides information on potential long-term (beyond 5 years) hormonal therapies. The EndoPredict^®^ score can be combined with tumor size and node status to obtain the more comprehensive EPclin risk score. This test can be used to guide the therapy decision for chemotherapy and extended endocrine therapy [52]. This multigene test was evaluated in the GEICAM-9906 trial as an independent prognostic parameter in patients with ER-positive, HER2-negative, and node-positive breast cancer for adjuvant chemotherapy and endocrine therapy. In the ABCSG6 and ABCSG8 trials, EndoPredict^®^ and EPclin were shown to provide additional information on the distant recurrence risk in patients with node-negative and node-positive disease, independent of clinicopathological parameters. EPclin can also be used to guide decision-making for the use of systemic chemotherapy in post-menopausal patients with ER-positive, HER2-negative, and node-negative breast tumors. For node-positive patients, EndoPredict^®^ clinical use is not recommended at this time.

### *BRCA 1/2* gene mutations and homologous recombination deficiency

Early breast cancer HER2-negative patients with high risk of recurrence should be tested for germline BRCA1 and BRCA2 mutations. For patients who have had previous surgery, high risk is defined as a tumor size >2 cm or any involved axillary node in TNBC cases, or ≥4 axillary nodes in HR-positive disease. In patients who have had NAT, high risk derives from either any residual cancer in TNBC or high-grade residual disease in HR-positive disease (defined as a Clinical Pathological State + ER Grade [CPS + EG] score ≥3).

The OlympiA study was a phase III, double-blind, randomized trial involving patients with HER2-negative early breast cancer with BRCA1 or BRCA2 germline pathogenic or likely pathogenic variants and high-risk clinicopathological factors who had received local treatment and neoadjuvant or adjuvant chemotherapy. Patients were randomly assigned to 1 year of oral olaparib or placebo. The primary endpoint was invasive disease-free survival. Olaparib treatment was associated with significantly longer survival free of invasive or distant disease than placebo [53]. One year of adjuvant olaparib is currently indicated either alone or concurrently with endocrine therapy in early breast cancer with BRCA1- or BRCA2-mutated high-risk patients that have received local treatment and neoadjuvant or adjuvant chemotherapy.

Homologous recombination deficiency (HRD) may allow consideration of using DNA-damaging agents such as PARP inhibitors. To analyze the impairment of the homologous recombination pathway, specific mutations in homologous recombination repair genes other than *BRCA1* or *BRCA2*, such as *PALB2*, *ATM*, *CHEK2* and others, can be examined. The use of genomic scars, mutational signatures [54], or the development of functional tests can also be considered [55]. Currently, olaparib is not indicated in HRD cases, although HRD testing may be useful in the future.

### PD-L1

Programmed cell death-1 (PD-1) protein is an immune checkpoint inhibitor expressed on the surface of T cells, B cells, natural killer T cells, monocytes, and dendritic cells, but not resting T cells. PD-1 binds to two ligands, PD-L1 (B7-H1) and PD-L2 (B7-DC). Activation of PD-1 by PD-L1 or PD-L2 induces downregulation of T-cell activity, reduced cytokine production, T-cell lysis, and induction of tolerance to antigens. In solid tumors, the PD-1/PD-L1 inhibitory pathway can silence the immune system by increasing the expression of PD-L1 on the tumor cell surface [56, 57].

Currently, neoadjuvant therapy in patients with early TNBC would include the combination of chemotherapy with pembrolizumab, regardless of PD-L1 expression. In this setting, the combination of chemotherapy with pembrolizumab (KEYNOTE-522 trial) has showed evidence of efficacy [58, 59]. The Keynote 522 trial evaluated patients with stages II–III TNBC treated with 4 cycles of paclitaxel plus carboplatin with the addition of 4 cycles of either pembrolizumab (*n* = 784) or placebo (*n* = 390). Patients received postoperative pembrolizumab or placebo for up to 9 cycles. The primary endpoints of the study were pathological complete response (pCR) and event-free survival. The percentage with pCR was significantly higher among those who received pembrolizumab plus neoadjuvant chemotherapy (64.8%) than among those who received placebo plus neoadjuvant chemotherapy (51.2%). The benefits of pembrolizumab-chemotherapy with respect to pCR were similar in PD-L1-positive and PD-L1-negative subgroups, although only 97 (16%) of the 602 cases in the trial were PDL-1-negative. In a subsequent analysis, event-free survival was also improved in the combination group [60].

### Tumor-infiltrating lymphocytes

Breast cancer is an immunogenic tumor and, in the last few years, morphological evaluation of tumor-infiltrating lymphocytes (TILs) in breast cancer has been proposed as a potentially useful biomarker. It has been reported that every 10% increment of stromal lymphocytes is associated with a 16% reduction of risk of death in TNBC, and values around 30–50% have been proposed for potential de-escalation of chemotherapies in this type of breast cancer. In the case of HER2-positive tumors, values >20% have been proposed for potential de-escalation of trastuzumab [61]. In contrast, increased TILs seemed to be an adverse prognostic factor for survival in luminal HER2-negative breast cancer, suggesting a different biology of the immunological infiltrate in this subtype [62].

In 2014, the International TILs Working Group described a method to quantify TILs on hematoxylin and eosin-stained slides using light microscopy [63] (Table 2). Recently, to maximize inter-observer reproducibility, the International TILs working group has created a website (www.tilsinbreastcancer.org) in which free training is available, and reference images are provided to allow direct visual comparison [64]. This methodology has been subsequently applied in lymph nodes and metastatic tissues [61]. We strongly recommend the use of this tool for self-validation before starting routine reporting of TILs.

**Table 2:** Recommendations of the TILs Working Group for assessing TILs in breast cancer [63]

1. One section (4–5 µm, magnification ×200–400) per patient is considered to be sufficient. Full sections are preferred over biopsies (in pretherapeutic neoadjuvant setting, cores can be used); currently, no validated methodology has been developed to score TILs after neoadjuvant treatment
2. TILs should be reported for the stromal compartment (% stromal TILs). The denominator used to determine the % stromal TILs is the area of stromal tissue
3. TILs should be evaluated exclusively within the borders of the invasive tumour, excluding TILs around ductal carcinoma in situ or normal lobules and zones with artefacts, necrosis, hyalinization as well as the previous biopsy site
4. All mononuclear cells (including lymphocytes and plasma cells) should be scored, but polymorphonuclear leukocytes are excluded
5. A full assessment of average TILs in the tumor area should be used
6. It should be scored as a continuous variable that will allow categorization of different thresholds and more accurate statistical analyses

TILs: tumor-infiltrating lymphocytes. For more information and self-training: www.tilsinbreastcancer.org [64]

Emerging data suggest that TILs quantification can help clinicians to identify breast cancers with better response to PD-1/PD-L1 inhibition and better prognosis especially in TNBC. In the neoadjuvant setting, TILs are predictive of pCR with chemotherapy [65].

Although not a standard biomarker, we recommend quantifying and reporting TILs to add valuable information about the immune response associated with each tumor.

## Advanced-stage breast cancer

### PIK3CA

In a population of 824 cases of HR-positive, HER2-negative tumors, the prevalence of phosphatidylinositol-4,5-bisphosphate 3-kinase catalytic subunit alpha (PIK3CA) mutations was 31.4% [66]. Treatment with alpelisib–fulvestrant has been found to prolong PFS among patients with *PIK3CA*-mutated, HR-positive, HER2-negative, advanced breast cancer who had received endocrine therapy previously in the SOLAR-1 phase III clinical trial [67]. Currently, alpelisib is indicated in combination with fulvestrant for the treatment of post-menopausal women, and men, with HR-positive, HER2-negative, locally advanced or metastatic breast cancer with a *PIK3CA* mutation after disease progression following endocrine therapy as monotherapy.

Alpelisib monotherapy has also shown efficacy in heavily pretreated ER-positive breast cancer with *PIK3CA* mutations (30% response rate) [68]. It is interesting that a recent real-life data study of 233 patients prospectively registered in the French alpelisib Early Access Program opened to *PIK3CA*-mutant, HR-positive, HER2-negative ABC patients treated with alpelisib and fulvestrant, showed that patients had received a median number of 4 prior systemic treatments for ABC, including CDK4/6 inhibitor (97.4%), chemotherapy (77.3%), or everolimus (56.2%), respectively.

Furthermore, it has been reported at the 2023 San Antonio Breast Cancer Symposium that inavolisib, another PI3K inhibitor, was more effective than placebo when combined with palbociclib and fulvestrant in the first-line treatment of patients with *PIK3CA*-mutated, HR-positive, HER2-negative, locally advanced or metastatic breast cancer (phase III INAVO1209).

Therefore, patients with advanced hormone-sensitive breast cancer should have their tumors tested for PIK3CA mutations, considering that at least one-third of cases harbor PIK3CA mutations and may benefit from PI3K inhibitors.

### *BRCA 1/2* gene mutations

In advanced breast cancer, the phase III OlympiAD study of olaparib compared with physician’s choice of chemotherapy was conducted in patients with *BRCA* mutations and HER2 non-overexpressing metastatic breast cancer that had received ≤2 prior therapies in the advanced setting. PFS, the primary endpoint, was significantly prolonged with olaparib versus standard therapy (7.0 vs. 4.2 months). There were no differences in OS, either at the interim or the final analysis [69].

The phase III EMBRACA trial enrolled patients with *gBRCA1/2*-mutated HER2-negative advanced breast cancer. Patients received talazoparib or physician’s choice of chemotherapy. Median PFS was significantly longer in the talazoparib group than in the standard therapy group (8.6 months vs. 5.6 months), but there were no differences in OS both at the interim and the final analysis [70].

Olaparib and, in some countries, talazoparib, are indicated as single agents for previously treated breast cancer patients who have HER2-negative or HR-positive locally advanced or metastatic breast cancer with germline *BRCA1/2* mutations, or as first-line therapy when patients are not suitable for standard therapies.

*BRCA1/2* testing, therefore, can provide a therapeutic opportunity in advanced breast cancer with germline *BRCA1/2* mutations and should be performed in this setting, if PARP inhibitors are available.

### PD-L1

The randomized controlled trials IMpassion130 [71] and Keynote-355 [58] have demonstrated the benefit of anti-PD-1/PD-L1 agents atezolizumab and pembrolizumab, respectively, plus chemotherapy, in first-line metastatic TNBC, with PFS and OS improved in PD-L1-positive patients [59, 71, 72]. Therefore, measurement of PD-L1 levels is a critical component in predicting patient benefit.

The combination of atezolizumab and nab-paclitaxel is recommended in the European Union as first-line treatment for PD-L1-positive (≥1%) metastatic TNBC, based on the results of the phase III IMpassion130 trial.

In the KEYNOTE-355 trial, with a more robust statistical design and results, 847 patients with advanced (unresectable, locally advanced, or metastatic) TNBC were randomly assigned to receive chemotherapy (nab-paclitaxel, paclitaxel or gemcitabine plus carboplatin) plus pembrolizumab or chemotherapy plus placebo. OS improved in patients who received pembrolizumab and had tumors with relatively high levels of PD-L1 protein (PD-L1 CPS of at least 10%): 23.0 months versus 16.1 months in control patients who received chemotherapy alone [73].

Patients with advanced TNBC should have PD-L1 tested in tumor tissue. According to the PD-L1 test that showed positive, pembrolizumab or atezolizumab should be used. The KEYNOTE studies used the 22C3 PD-L1 immunohistochemistry assay (Agilent, Carpinteria, CA) to calculate a CPS estimated as the ratio of PD-L1-positive cells (tumor cells plus immune cells divided by the total number of viable tumor cells ×100 with a cut-off ≥10 [59, 73]). The IMpassion trials, in contrast, used the SP142 PD-L1 immunohistochemistry assay (Ventana, Tucson, AZ), measuring the proportion of tumor area that is occupied by PD-L1 staining in IC with cut-off in >1% of staining [58, 71, 72].

Most laboratories do not regularly use all possible PD-L1 antibodies, and harmonization studies are needed. Rugo, et al. compared PD-L1 status on IC (VENTANA SP142, SP263, Dako 22C3) or as a CPS, and concluded that 22C3 and SP263 identified more patients as PD-L1-positive than SP142 [74].

### *ESR1* mutations

A key mechanism of endocrine resistance is through missense mutations in *ESR1*, the gene that encodes for ER alpha. *ESR1* mutations are present in about 30% of patients with metastatic breast cancer who have received aromatase inhibitors, although only in 5% of breast cancer recurring after adjuvant aromatase inhibitors, and 1% of endocrine therapy-naïve metastatic breast cancer [75]. Ligand binding domain mutations of *ESR1* make the receptor constitutively active and thus unaffected by aromatase inhibitor depletion of estrogen. In contrast, *ESR1* mutations do not appear to be a main mechanism of resistance to tamoxifen or fulvestrant [76]. Several selective ER modulators or covalent antagonists are being tested specifically against *ESR1* mutations. Elacestrant is one of these oral selective ER degraders. The randomized phase III EMERALD trial enrolled patients with ER-positive, HER2-negative advanced breast cancer who had one or two lines of endocrine therapy, required pre-treatment with a cyclin-dependent kinase 4/6 inhibitor, and had one or no lines of chemotherapy. Primary endpoints were PFS by blinded independent central review in all patients and patients with detectable *ESR1* mutations. Detectable ESR1 mutations in circulating tumor DNA were detected in 47.8% of patients. PFS was prolonged in all patients, particularly in patients with *ESR1* mutation. Elacestrant monotherapy is indicated by the Food and Drug Administration (FDA) and European Medicines Agency (EMA) for the treatment of post-menopausal women and adult men with HER2-negative, ER-positive advanced breast cancer, with an *ESR1*-activating mutation who have disease progression after receiving at least one line of endocrine therapy including a CDK 4/6 inhibitor. Elacestrant is becoming increasingly available, and testing for *ESR1* mutations in advanced breast cancer with either liquid or tissue biopsy is currently recommended by clinical guidelines [41, 77].

### AKT pathway activation

An additional mechanism of endocrine resistance in advanced breast cancer is related to serine/threonine kinase (AKT). AKT is the key node of the PI3K–AKT–PTEN signaling pathway. Overactivation of the pathway occurs in approximately half of HR-positive, HER2-negative breast cancers by means of activating mutations in *PIK3CA* and *AKT1* and inactivating alterations in *PTEN*.

Capivasertib is an orally bioavailable, small molecule inhibitor of AKT. In a randomized, double-blind, placebo-controlled, phase III trial, 708 patients were assigned to receive either oral capivasertib plus fulvestrant or a matching placebo plus fulvestrant. Capivasertib–fulvestrant therapy resulted in significantly longer PFS than treatment with fulvestrant alone among patients with HR-positive advanced breast cancer whose disease had progressed during or after previous aromatase inhibitor therapy with or without a CDK4/6 inhibitor. AKT pathway alterations (*PIK3CA*, *AKT1*, or *PTEN*) were assessed in tumors. Similar results were observed in the overall population and in the AKT pathway altered population. At this point, AKT pathway testing for capivasertib use is still in research [78].

### NTRK

*NTRK* gene fusions are tumor-agnostic biomarkers that predict response to *NTRK* inhibitors. Secretory breast carcinoma is a special histological type of breast carcinoma that carries the *NTRK3*-*ETV6* fusion in about 90% of cases. Detection of this fusion by fluorescence ISH (FISH) or NGS can help in the correct diagnosis of this entity and in predicting response to inhibitors in advanced tumors. In contrast, less than 1% of all breast cancer cases harbor *NTRK* fusions. Currently, *NTRK* testing is not required in advanced breast carcinoma.

### TROP-2

TROP-2 is a transmembrane calcium protein belonging to the EpCAM family that is expressed by normal human multistratified epithelia and trophoblast cells. Overexpression can be present in several solid tumors, including TNBC. Approximately 86% of TNBCs are TROP-2-positive [79].

Sacituzumab govitecan is an antibody–drug conjugate that combines a humanized monoclonal antibody binding to TROP-2-expressing cancer cells (sacituzumab) with a topoisomerase I inhibitor (govitecan). Sacituzumab govitecan as a single agent is indicated for the treatment of adult patients with unresectable or metastatic TNBC who have received two or more prior systemic therapies, with at least one for advanced disease [80].

Different levels of TROP-2 expression did not have an apparent effect on the efficacy of the treatment in this study. Currently, TROP-2 testing is not required in order to use sacituzumab govitecan for this indication.

### Other biomarkers

Assessment of other biomarkers that are targets of novel therapies, such as *CDK* amplification, *FGFR1* amplification, or *PTEN* loss of heterozygosity or mutations, is not currently recommended.

## Biomarker assessment following NAT

NAT, including chemotherapy, anti-HER2 therapy and hormonal therapy administered before surgery, has become part of the standard-of-care treatment of patients not only with locally advanced breast cancer but also with operable tumors, particularly in HER2-positive carcinomas and TNBC. Besides reducing tumor burden, NAT provides a unique opportunity to evaluate the tumor response to different treatments. The pCR is a well-established surrogate marker of improved prognosis in breast cancer. However, not all patients obtain a pCR, and these patients have a varying risk of relapse [81]. Importantly, substantial biological differences exist between treatment-naive breast cancer and residual tissue following NAT [82]. Re-assessment of biomarkers in the residual breast cancer tissue may have both prognostic and potential therapeutic implications. Post-NAT pathological stage and biomarker status may help guide adjuvant treatment decisions. Accordingly, this SEOM-SEAP consensus guideline recommends the following biomarker determinations, depending on the type of NAT.

### Following neoadjuvant chemotherapy with or without anti-HER2 therapy

Classic histopathological parameters such as *ypTNM* classification and histological grade provide valuable prognostic and predictive information when assessed in residual breast cancer tissue after NAT [83]. Additionally, the Residual Cancer Burden (RCB) index [81] is a clinically validated and standardized reporting system that does not incorporate response, but establishes risk of recurrence in patients with residual disease in both the breast and the lymph nodes. The parameters to be quantified and reported, as well as a calculator, are available online [84].

IHC-based biomarkers such as ER, PR, and HER2 may also be re-assessed if negative prior to treatment, to allow patients to benefit from targeted therapies and to obtain biological explanation for possible causes of intrinsic resistance to treatments. Although clinical decisions are not still made based on TILs, immune markers are among the most promising biomarkers in the post-NAT setting, in which extensive tumor infiltration by lymphocytes indicates a good prognosis in some breast tumor types, irrespective of residual tumor size. In TNBC, TILs levels are significantly associated with improved recurrence-free survival (RFS) and OS and add further prognostic information to RCB class, particularly in RCB class II [85].

### Following neoadjuvant endocrine therapy in post-menopausal patients

#### Ki-67 after short-term endocrine treatment

Ki-67 expression after 2 weeks of treatment (Ki-67_2W_) may improve the prediction of RFS better than Ki-67 at baseline (Ki67_B_), as observed among patients enrolled in the IMPACT trial [86]. The POETIC trial provided evidence for the clinical validity of on-treatment aromatase inhibitor Ki-67_2W_ in addition to Ki-67_B_ to predict those with high residual risk of recurrence in spite of standard-of-care therapy [87]. Patients whose Ki-67_B_ was low had good results, with 85% of those receiving endocrine therapy alone. Patients whose tumors had a high baseline Ki-67 and a low Ki-67_2w_, had a better prognosis at 5 years than those who continued to have a high Ki-67_2W_.

#### Preoperative endocrine prognostic index (PEPI score)

Multivariable testing of post-treatment tumor characteristics including pathological tumor size, node status, Ki-67 level, and ER status were independently associated with RFS and breast cancer-specific survival. Of note, patients with low pathological stage at surgery and a favorable biomarker profile (preoperative endocrine prognostic index [PEPI] score 0) had such a low rate of recurrence that further adjuvant systemic therapy beyond continuation of endocrine therapy seemed unnecessary. In contrast, patients with high pathological stage disease at surgery and a poor PEPI score (PEPI group 3) had a significantly higher risk of relapse, and therefore should be offered appropriate adjuvant treatments [88].

## New technologies

### Next-generation sequencing (NGS)

At present, NGS in breast cancer remains a research tool. NGS should not be used as an exploratory tool to prescribe treatments beyond the indications for which they have been approved, regardless of whether the target genetic alteration is detected [89]. The SAFIR01 study identified targetable genomic alterations in 195 cases (46%) in a series of 423 patients with advanced breast cancer, although ultimately only 55 received personalized treatment and just 13 showed any type of response [90].

Therefore, more research is needed into the role of NGS in clinical practice before incorporating it into routine use, and efforts should be made to educate clinicians to increase their knowledge and confidence in such technologies [91]. An expert panel has produced a guidance document to rank DNA alterations into tiers of evidence for clinical utility for selecting breast cancer patients for targeted therapies according to ESMO Scale for Clinical Actionability of molecular Targets (ESCAT) [92]. Among 40 recurrent driver alterations described in breast cancer, only HER2 amplification, germline *BRCA1/2* mutations, and *PIK3CA* mutations were given level 1A evidence as molecular targets whereas *NTRK* fusions and microsatellite instability (MSI) were ranked as 1C evidence.

 An update of the ESMO recommendations for the use of NGS in advanced cancer has been published very recently [93]. The authors consider that, since NGS can substitute germline *BRCA* testing in most patients and ESR1 mutations have been reclassified as level 1A, performing NGS in advanced breast cancer (tumor or plasma) is recommended in patients with HR-positive/HER2-negative disease after resistance to endocrine therapy.

### Liquid biopsy, circulating tumor cells, and circulating tumor DNA

Traditional methods of cancer detection, such as tissue biopsy, can sometimes not be comprehensive enough to capture the entire genomic landscape of breast tumors. The role of liquid biopsy in cancer management has been gaining increased prominence in the past decade. Various components of tumor cells released into the blood circulation can be analyzed in liquid biopsy sampling, some of which include circulating tumor cells (CTCs), circulating tumor DNA (ctDNA), cell-free RNA, tumor-educated platelets, and exosomes. These components can be used for different purposes. Currently one of the most investigated utilities of liquid biopsy is ctDNA testing in advanced breast cancer. The recent advances in massively parallel sequencing technologies have empowered liquid biopsies, particularly ctDNA analysis, to be the new paradigm in personalized cancer management.

Plasma ctDNA detection may overcome some of the current limitations in tumor tissue procurement and serves as a convenient and non-invasive method to capture tumor heterogeneity and genetic evolution along patients’ cancer journeys. ctDNA can be sequenced for genetic profiling of the tumors in selected patients for mutation-directed therapy [94–96]. Data from the plasmaMATCH study were recently reported [97], and showed that ctDNA testing for mutations has high sensitivity and accuracy for widespread adoption in clinical practice even to screen for rare oncogenic mutations. It is efficient and rapid for screening and allows evaluation of different acquired mutations throughout the evolution of the disease.

Recently, the FDA approved the Therascreen PIK3CA RGQ polymerase chain reaction assay as a companion diagnostic assay to detect *PIK3CA* mutations in breast cancer for both tissue and liquid biopsies, bringing the role of liquid biopsy into breast cancer management. In this context, alpelisib, a *PI3K* inhibitor, was the first agent to be approved by the FDA and EMA [98].

In breast cancer, the current clinical application of ctDNA includes detection of drug-resistant clones. In this way *ESR1* mutation detection has already been shown as a predictive biomarker, used in clinical practice for metastatic hormone receptor breast cancer and in some cases, early switch of hormone therapy [99]. *ESR1* mutations assessment using liquid biopsy (by digital-PCR or NGS) [100], is a current standard accepted by clinical guidelines [41, 77], as has been described earlier in this document.

CTC plasma count analysis after curative tumor resection surgery may facilitate early detection of minimal residual disease, aiding in the initiation of adjuvant therapy to prevent recurrence. Furthermore, CTC plasma count can be used for monitoring disease response, detecting and predicting risk of progression or relapse [101].

In the field of breast cancer, liquid biopsy has been a research hot-spot in recent years, playing a key role in monitoring breast cancer metastasis, predicting disease recurrence, and assessing clinical drug resistance. Liquid biopsy has the advantages of non-invasive, high sensitivity, high specificity, and real-time dynamic monitoring. While clinical application is not yet a reality, the research prospects of CTCs and cfDNA in breast cancer are worth exploring and discovering.

Researchers and clinicians are currently working to validate the clinical utility of ctDNA in diagnostics, prognostics, the surveillance of minimal residual disease, and the monitoring of therapeutic response [94–96].

### MSI and MMRd

High microsatellite instability (MSI-H)/Mismatch repair deficiency (MMRd) is considered a tumor-agnostic biomarker that predicts response to ICI in some advanced cancers [102]. However, the frequency of MSI-H/MMRd is very low among breast carcinomas, and currently it is not a recommended biomarker. While it is controversial whether women with Lynch syndrome have an increased risk of developing breast carcinomas, about 50% of breast carcinomas in patients with Lynch syndrome carry MMRd. Immunohistochemistry is the most frequently used method to detect MMRd, although other molecular methods can be used.

### Tumor mutational burden

Tumor mutational burden (TMB) may be a good biomarker for the indication of immune checkpoint inhibitors, as it can reflect a high neoantigen burden, which can lead to an increased immune response. Moreover, the FoundationOne^®^ CDx assay has been approved as a companion diagnostic for tumor-agnostic pembrolizumab in patients with a TMB of ≥10 mutations per megabase. However, not all immune checkpoint inhibitors show the same correlation with TMB (i.e., atezolizumab) and not all assays have the same thresholds. There is a need for harmonization, and care should be taken when interpretating TMB for specific treatments [103, 104].

## Conclusions

**Fig. 2:**
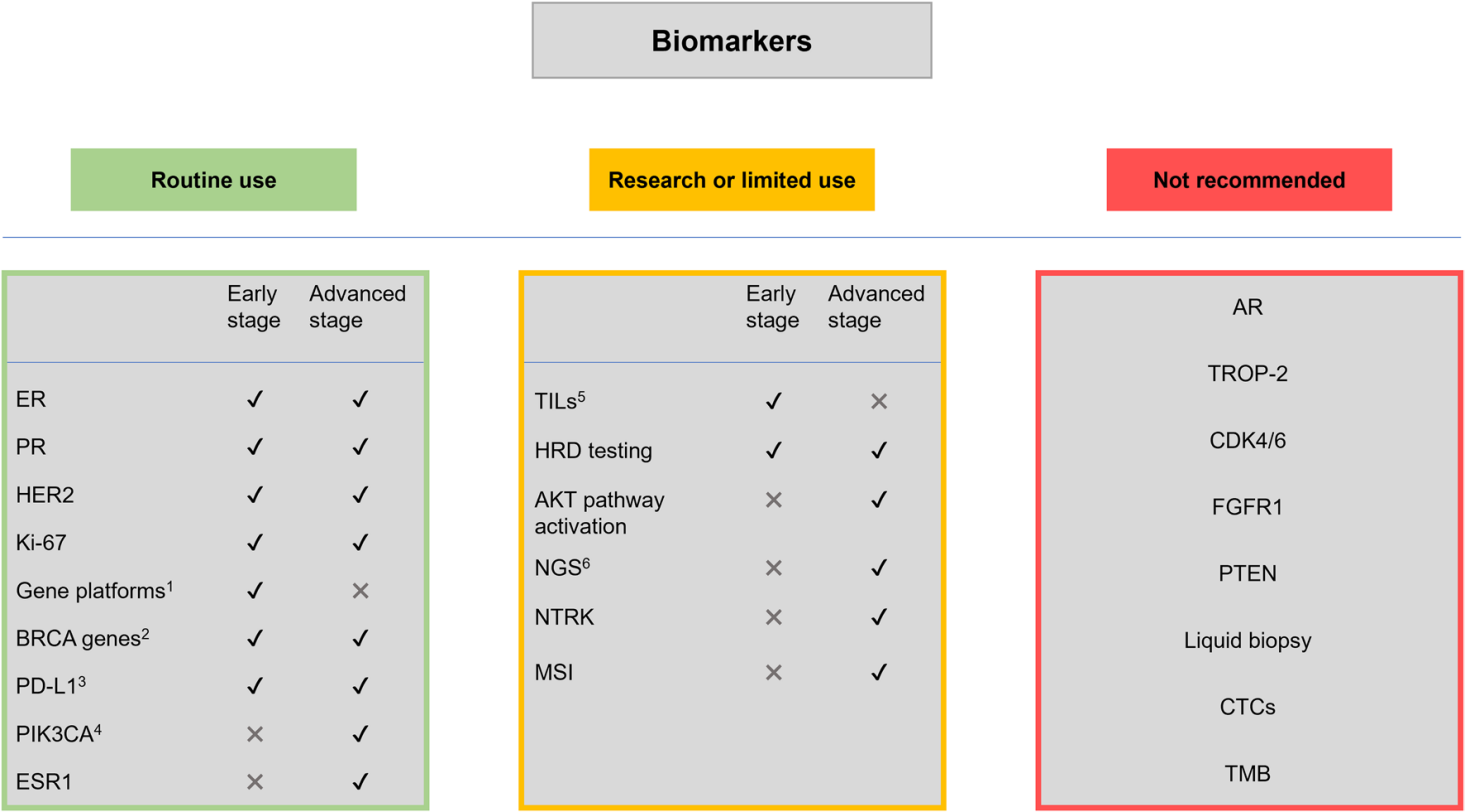
Current routine use, research use, and not-recommended use of biomarkers for breast cancer. AKT: serine/threonine kinase; AR: androgen receptor; BRCA: breast cancer; CTCs: circulating tumour cells; ER: estrogen receptor; ESR1: estrogen receptor 1; FGFR1: fibroblast growth factor receptor 1; HER2: human epidermal growth factor receptor 2; HRD: homologous recombination deficiency; MSI: microsatellite instability; NGS: next-generation sequencing; NTRK: neurotrophic receptor tyrosine kinase 1; PD-L1: programmed death ligand 1; PIK3CA: phosphatidylinositol 4,5-bisphosphate 3-kinase catalytic subunit alpha; PR: progesterone receptor; PTEN: phosphatase and tensin homolog; TILs: tumour-infiltrating lymphocytes; TMB: tumor mutational burden; TROP-2: tumor-associated calcium signal transducer 2. ^1^Mammaprint^®^, Oncotype DX^®^, Prosigna^®^ or EndoPredict^®^ in early luminal breast cancer with low risk of recurrence; ^2^In advanced triple-negative or luminal breast cancer; ^3^In advanced triple-negative breast cancer; ^4^In advanced luminal breast cancer; ^5^Some studies relate them to responses to neoadjuvant chemotherapy, ^6^Approved in the United States of America as a companion diagnostic to a *PI3K* inhibitor

To plan adequate therapy in patients with early breast cancer (Fig. 2), pathology reports should include in all cases the expression and levels of ER, PR, HER2, and Ki-67, in addition to histological grade (as well as *BRCA* in high-risk HR-positive, HER2-negative patients, and TNBC) to assist prognosis and to establish current therapeutic options available, including hormone therapy, chemotherapy, anti-HER2 therapy and PARP inhibitors. In ER-positive HER2-negative early breast cancer patients, one of the several available genetic prognostic platforms (Oncotype DX^®^, MammaPrint^®^, Prosigna^®^, or EndoPredict^®^) may be used to establish prognosis and to discuss with the patient whether adjuvant treatment may be limited to hormonal therapy.

In advanced breast cancer, physicians should have available (in addition to ER, PR, Ki-67 and HER2) the results for *BRCA* and PI3K in HR-positive, HER2-negative cases, ESR1 in ER-positive, HER2-negative cases after progression of first-line hormonal therapy including a CDK inhibitor, and PD-L1 in TNBC.

Newer biomarkers and technologies including TILs, HRD testing, AKT pathway activation, and NGS are experimental at this point. Other biomarkers such as NTRK or MSI may be useful in a limited subset of advanced breast carcinomas although are not standard tests at this point.

## Data Availability

Not applicable.
